# Inside the Outbreak of the 2009 Influenza A (H1N1)v Virus in Mexico

**DOI:** 10.1371/journal.pone.0013256

**Published:** 2010-10-08

**Authors:** Hector M. Zepeda-Lopez, Lizbeth Perea-Araujo, Angel Miliar-García, Aarón Dominguez-López, Beatriz Xoconostle-Cázarez, Eleazar Lara-Padilla, Jorge A. Ramírez Hernandez, Edgar Sevilla-Reyes, Maria Esther Orozco, Armando Ahued-Ortega, Ignacio Villaseñor-Ruiz, Ricardo J. Garcia-Cavazos, Luis M. Teran

**Affiliations:** 1 Laboratorio de Medicina de Conservación, Escuela Superior de Medicina, Instituto Politécnico Nacional, Distrito Federal, Mexico; 2 Departamento de Biotecnología y Bioingeniería, Centro de Investigación y de Estudios Avanzados, Instituto Politécnico Nacional, Distrito Federal, Mexico; 3 Inmunogenetica y Alergia, Instituto Nacional de Enfermedades Respiratorias, Distrito Federal, Mexico; 4 Dirección General, Instituto de Ciencia y Tecnología, Distrito Federal, Mexico; 5 Dirección General, Secretaria de Salud, Distrito Federal, Mexico; 6 Subdirección, Secretaria de Salud, Distrito Federal, Mexico; University of Pretoria, South Africa

## Abstract

**Background:**

Influenza viruses pose a threat to human health because of their potential to cause global disease. Between mid March and mid April a pandemic influenza A virus emerged in Mexico. This report details 202 cases of infection of humans with the 2009 influenza A virus (H1N1)v which occurred in Mexico City as well as the spread of the virus throughout the entire country.

**Methodology and Findings:**

From May 1st to May 5th nasopharyngeal swabs, derived from 751 patients, were collected at 220 outpatient clinics and 28 hospitals distributed throughout Mexico City. Analysis of samples using real time RT-PCR revealed that 202 patients out of the 751 subjects (26.9%) were confirmed to be infected with the new virus. All confirmed cases of human infection with the strain influenza (H1N1)v suffered respiratory symptoms. The greatest number of confirmed cases during the outbreak of the 2009 influenza A (H1N1)v were seen in neighbourhoods on the northeast side of Mexico City including Iztapalapa, Gustavo A. Madero, Iztacalco, and Tlahuac which are the most populated areas in Mexico City. Using these data, together with data reported by the Mexican Secretariat of Health (MSH) to date, we plot the course of influenza (H1N1)v activity throughout Mexico.

**Conclusions:**

Our data, which is backed up by MSH data, show that the greatest numbers of the 2009 influenza A (H1N1) cases were seen in the most populated areas. We speculate on conditions in Mexico which may have sparked this flu pandemic, the first in 41 years. We accept the hypothesis that high population density and a mass gathering which took in Iztapalapa contributed to the rapid spread of the disease which developed in three peaks of activity throughout the Country.

## Introduction

In 2009, human infection with the influenza A (H1N1)v virus became a health burden throughout the world. Initial cases were seen in the town of La Gloria in the Eastern coastal region of México in the state of Veracruz, Similar cases of influenza A (H1N1)v virus occurred shortly thereafter in other parts of Mexico including Oaxaca, Mexico City and San Luis Potosi [1]. On April 23rd, several cases of severe respiratory illness were confirmed as being of swine origin consisting of an A/H1N1 virus [2]. On May 1st, the Mexican Secretariat of Health (MSH) reported that the largest number of influenza confirmed cases were located in the Federal District (57% of total cases), also known as Mexico City [3]. To date however, the origin of the virus has not been determined. Using phylogenetic analysis, Smith et al. [4] showed that the new influenza A (H1N1)v has been circulating in swine for at least 10 years based on the finding that common ancestor of the 2009 influenza A (H1N1)v arose between 9.2 and 17.2 years ago: the outbreak strain resulted from a reassortment of two previously circulating strains: a “triple reassortant” that has been circulating in North America since 1998 and an H1N1 strain that has been circulating for decades in swine populations in Europe and Asia. It has been proposed that mutations in the hemagglutinin (HA) and neuraminidase (NA) proteins could have facilitated human to human transmission to the 2009 influenza A (H1N1)v virus, over time [5], [6]. Interestingly, Itoh et al. have reported that pathogen-free pigs inoculated with the 2009 influenza A (H1N1)v (A/calfornia/04/09 H1N1) did not develop influenza like-illness despite the virus replicating efficiently in the respiratory airways [7]. More recently, Bi et al have detected several influenza A virus subtypes such as H1N1 (including the novel H1N1v) and H3N2 in apparently healthy domestic pigs [8].Thus, asymptomatic infection in pigs is consistent with the lack of detection over an extended period of time. The first case of human infection by a triple reassortant was seen in United States in December 2005 and only 11 cases were reported subsequently until February 2009 [9]. As of February 24^th^, 2010 the MSH has reported a total of 70866 confirmed influenza A (H1N1)v cases and 1076 deaths throughout Mexico [10]. Concurrently, the new influenza A(HIN1) virus was spreading globally to over 212 countries and has to date caused 16226 deaths worldwide [11]. On 11 June 2009, the World Health Organisation (WHO) declared this outbreak to be the first flu pandemic in 41 years.

Most previous studies investigating the outbreak of the 2009 A(H1N1)v influenza virus in Mexico have focused on studying critically ill patients: Chowell et al reviewed 2155 records from the Mexican National Epidemiological Surveillance System (SINAVE) between March 24th and 29^th^ 2009 and reported an increased rate of severe pneumonia in patients between 5 and 59 years [12]. In parallel, Perez-Padilla et al. reported 18 cases of pneumonia, 7 of who died between March 24th and April 24th, among 98 patients hospitalized for acute respiratory disease [13]. Subsequently, a study conducted in 6 hospitals between March 24^th^ and June 1, 2009, showed that 58 infected out of 899 hospitalized patients developed critical illness [14]. In a larger study conducted at the Instituto Mexicano del Seguro Social by Echevaria-Zuno et al. reported 6945 cases with the 2009 A(H1N1) influenza virus with <1% who died and 7% were hospitalized [15].

The present study describes 202 cases of influenza A (H1N1)v which occurred early during the outbreak of this novel virus in Mexico City and its eventual spread throughout Mexico and discuss it in the context of a mass gathering of 2 million people which took place in Iztapalapa, Mexico City, during the Easter season (5–11 of April, 2009). It is well established that mass gatherings present a particular challenge for public health, with unusual population increase, high crowd density, visitors, temporary catering and accommodation facilities contributing to an increased in the transmission of communicable diseases [16]. A previous study conducted during a mass gathering of people in Sydney Australia [17], held from 15 to 20 July 2008 (the Sydney World Youth day), showed that undertaking appropriate preparedness plans reduces substantially the impact of influenza virus infection. We hypothesize that the mass gathering which took place in Iztapalapa could have contributed to the spread on the virus.

## Methods

### Subjects

Seven hundred and fifty one individuals, who sought medical care from May 1st to May 5^th^ at 220 outpatient clinics and 28 hospitals distributed throughout Mexico City, participated in this study. Nasophariyngeal-swab samples were taken from patients upon arrival at the clinics. Specimens were homogenized in virus transport medium and kept on ice before diagnosis. The age range of the subjects was from 5 to 70 years. The study was approved by the Superior School of Medicine, Instituto Politécnico Nacional Ethical Committee and written informed consent was obtained from all subjects.

### Detection of the 2009 Influenza A (H1N1)v Virus

In order to make a rapid diagnosis, specific primers and probes designed by TIB MOLBIOL (GmbH Eresburgstr; Berlin) were used for specific detection of influenza A (H1N1)v. Quantitative real time PCR primers were designed based on the conserved matrix protein gene (MP) following WHO recommendations of 2007–08 [18]. This assay had been previously tested on clinical samples at the Universities of Bonn, Marburg, Berlin (Robert Koch Institute [RKI]) and Hamburg (Bernhard-Nocht-Institute [BNI]) www.rki.de/cln_091/nn_200120/DE/Content/InfAZ/I/Influenza/IPV/Schweinegrippe_PCR.html). Further detection and confirmation was carried out according to CDC and WHO guidelines. Both assays detected the influenza (H1N1)v in all 202 cases.

### Data

The time location series of 2009 influenza A H1N1 virus were extracted from official reports provided by the MSH (www.dgepi.salud.gob.mx/influenza/AH1N12009/boletines_index.html). Each report contained the following clinical and demographic data: number of cases, symptoms, deaths, province, and district. Information on hospitalization was incomplete (e.g. reports from September to December lacked full information on hospitalization). These data were collected by the SINAVE which is a surveillance system created in 1995 to detect new diseases in Mexico (www.dgepi.salud.gob.mx/sinave/sinave_02.html). To display the outbreak magnitude and trend over time, epidemic curves were constructed by considering weekly numbers from April to December 2009.

### Statistical Analysis

Variables of the outbreak, including clinical variables and geographic distribution, were illustrated using graphs, histograms and colour coded, density maps (maps were drawn using the software Photoshop). We estimated rates of clinical infection (attack rate), hospitalization and deaths (case-fatality rate) with a 95% confidence interval. This analysis was applied to all Mexican States. The Chi square test was used for comparisons. Statistical analysis was performed using the SPSS 16.0 software for windows (SPSS, Inc.; Chicago, IL).

## Results

### Clinical symptoms

Analysis of nasopharyngeal-swabs using real time RT-PCR confirmed that 202 patients out of the 751 (29.3%) were infected with influenza A (H1N1)v. All confirmed cases of human infection with the strain influenza (H1N1)v, presented with respiratory symptoms including fever, cough, headache, runny nose, muscle pain, sore throat, chills, nasal obstruction, conjunctivitis and abdominal pain (supplementary figure S1). Twelve patients required hospitalization, of whom two died as a result of respiratory failure. The age of patients with confirmed 2009 influenza A(H1N1)v infection ranged from 3 months to 70 years: a total of 32% were between the ages of 5 and 19 years, 28% between 20 and 39 years, 18% between 40 and 59 years and 3% were 60 years of age or older (supplementary figure S1).

### Distribution of confirmed cases with 2009 influenza A(H1N1)v

**Figure 1:**
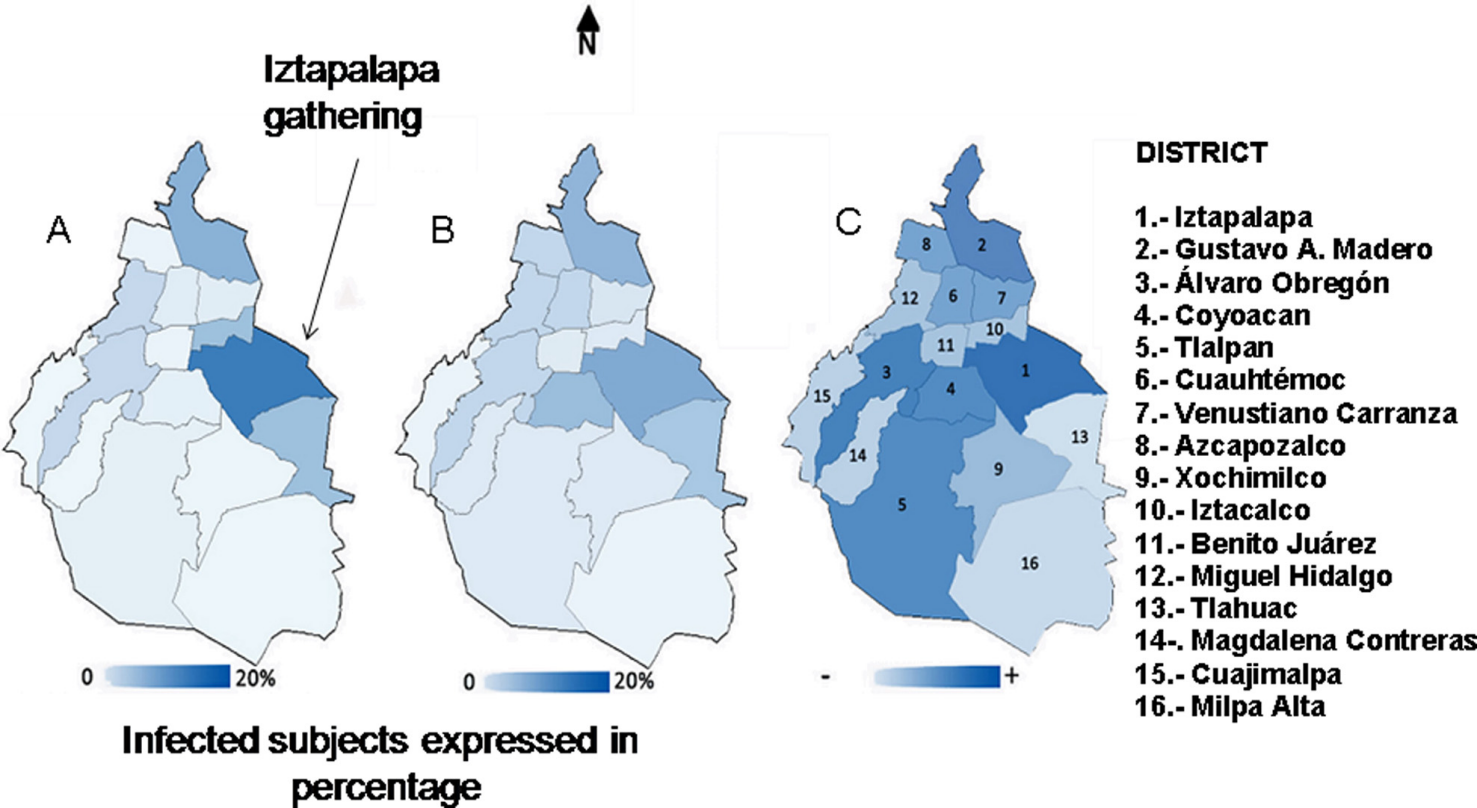
Destribution of cases. A) distribution of 202 confirmed cases of human infection with 2009 influenza A(H1N1)v in the Federal District, B) distribution of 515 cases reported by the Mexican Secretariat of Health as May the 5^th^. (http://www.dgepi.salud.gob.mx/influenza/AH1N12009/boletines/052009/Influenza_H1N1_Mexico_06may09.pdf, C) distribution of population in the Federal District per neighbourhood according to the 2005 census. 0 to 20% in the bottom represents the percentage of people infected with the novel virus in each district. C represents the population density in the Federal District (range 73625 [Cuajimalpa] to 1,820888 [Iztapalapa]).

Demographic analysis of data from this study (Fig 1A) agreed with those from the Mexican Secretariat of Health up to and including May 5^th^ (Fig 1B) and showed that the greatest number of confirmed cases of the 2009 influenza A (H1N1)v virus were seen in neighbourhoods on the northeast side of México City including Iztapalapa (n = 38), Gustavo A. Madero (n = 28), Iztacalco (n = 19), and Tlahuac (n = 19) while the lowest number of confirmed cases were observed in the Southwest of Mexico City.

### Spread of the 2009 influenza A(H1N1)v

**Figure 2:**
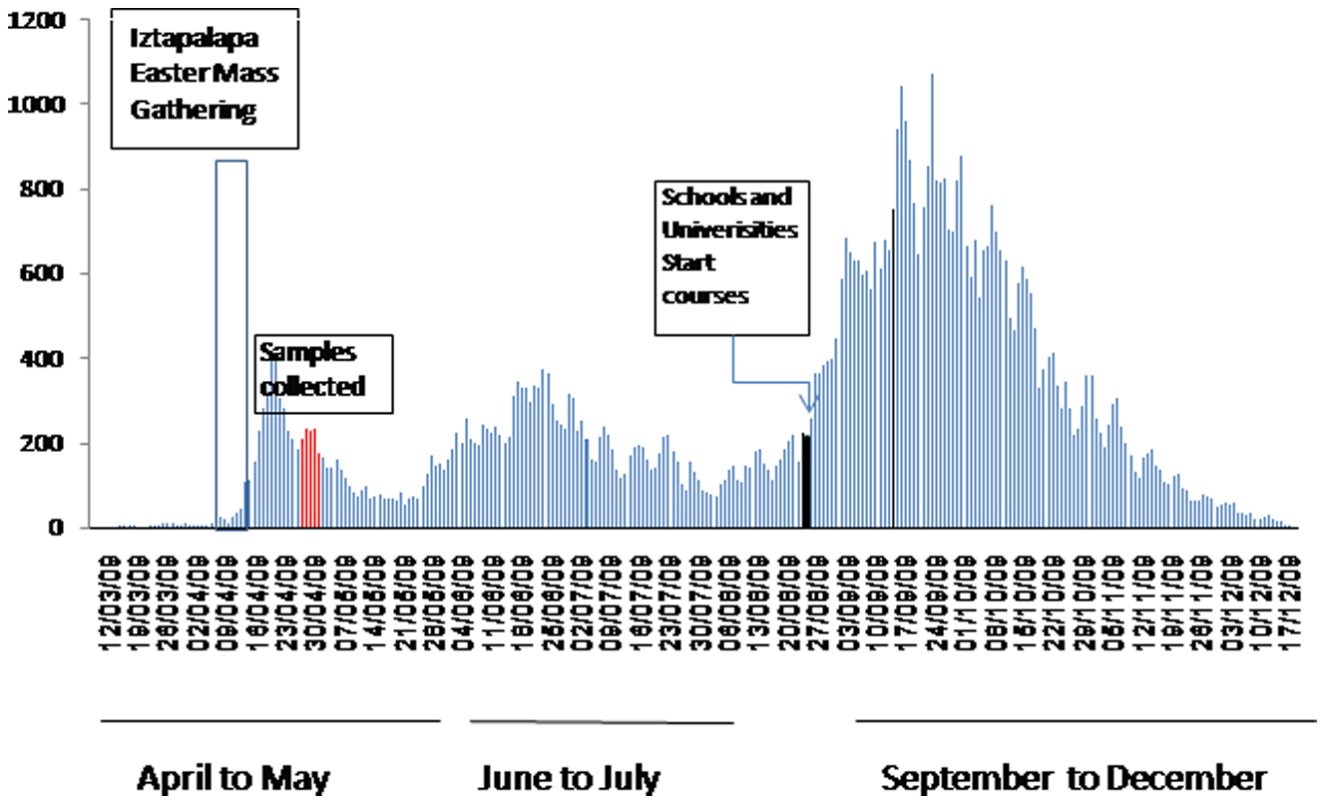
Epidemiological curve of confirmed cases of human infection during the outbreak of the 2009 influeza A(H1N1)v. Three waves of influenza activity were seen: the first wave occurred between April and May, the second wave took place between June and July and the third, between September and December. Bars in red show the days when samples were collected. Iztapalapa Play passion (Bar in white) and return to schools (Blue arrow). Text in white boxes describes each event.

**Figure 3:**
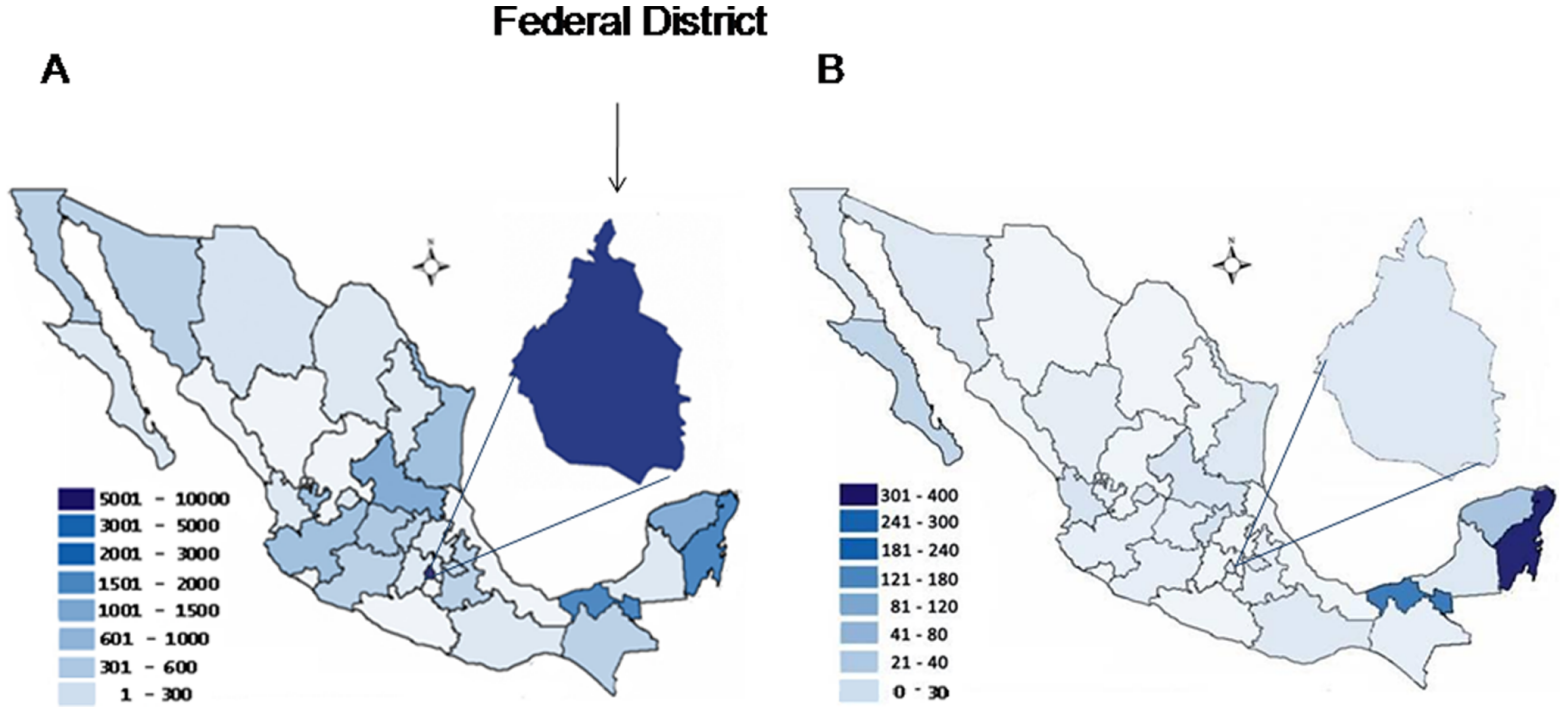
Distribution of human infection with 2009 influenza A(H1N1)v throughout México during the third wave. A shows absolute numbers of confirmed cases infected with the novel virus while B gives the estimated attack rate throughout México during the third wave. Data was obtained from the Sistema Nacional de Vigilancia Epidemiológica/Dirección General Adjunta de Epidemiología/Secretaría de Salud. www.dgepi.salud.gob.mx/influenza/AH1N12009/boletines_index.html, accessed 1 March 2010 (last time).

**Figure 4:**
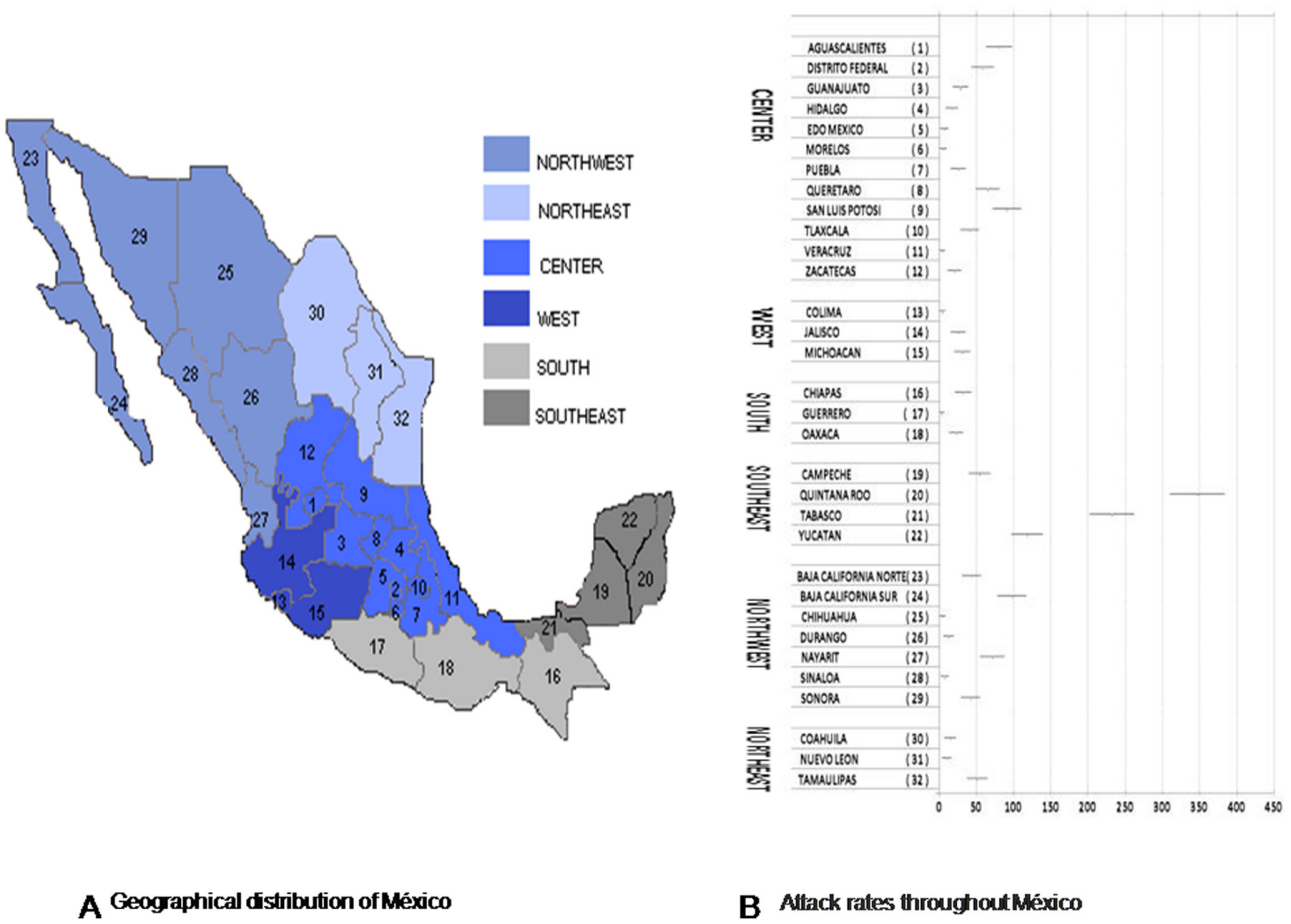
Estimated attack rate of influenza A (H1N1)v virus infection in the different Mexican States during the 3^rd^ wave. A. Map shows geographic distribution of cases throughout Mexico. B. Table shows distribution of cases quantitatively (lanes represent the mean [95% confidence interval]). Data was obtained from the Mexican Secretariat of Health: www.dgepi.salud.gob.mx/influenza/AH1N12009/boletines_index.html, accessed 1 March 2010 (last time).

In order to assess the spread of human infection with the 2009 influenza A(H1N1)v virus we have also analysed the whole data reported by the MSH which showed three waves of influenza activity (Fig 2). The first wave occurred between April and May with the highest attack rates of the novel influenza A localized to México City (106.4/100.000), followed by Jalisco (81.3/100.000), Tabasco (80.8/100.000), and San Luis Potosi (65.6/100.000). The corresponding attack rate for Quintana Roo was 42.4/100.0000. The second wave took place between June and July with the majority of cases seen in South of Mexico mostly within the states of Yucatan, Quintana Roo, Tabasco and Chiapas with 365/100.000, 262/100.000, 205/100.000 and 105.3/100.000, respectively, while the attack rate in Mexico City decreased to 35.5/100.000. During the third wave, between September and December, the greatest number of cases were still reported in Quintana Roo (347/100.00), Tabasco (232.2/100.000) and Yucatan (118.4/100.000). At this point the attack rate in Mexico City was 58,3/100.000. Figures 3 and 4 show the distribution of cases and attack rate of the novel virus throughout México during the 3^rd^ wave.

The estimated hospitalization rate for the first and second wave was 73/1000 and 102/1000, respectively. There was not information available for the 3^rd^ wave. However, by reviewing the number of deaths reported by the Mexican Secretariat of Health, the case-fatality rate (CFR) was calculated: the virus killed 0.18% and 0.11% during the first and second waves, respectively. However, this increased to 1.88% during the third wave (p≤0.001 in comparisons with either the 1^st^ or 2^nd^ waves).

## Discussion

This study described the early outbreak of the novel influenza A (H1N1)v virus in Mexico City and showed that the greatest number of confirmed cases with this novel virus were seen in the most populated districts. For example, the districts Iztapalapa and Gustavo A Madero, which had the highest incidence of cases, are densely populated with almost 2 million- and 1.2 million people, respectively [19]. The demonstration that the 515 cases reported by the Secretariat of Health showed a similar pattern of distribution confirms that densely populated areas are susceptible to influenza transmission. The mechanisms by which people get infected are not fully understood, however, potential modes of influenza transmission include direct contact with infected individuals, exposure to virus contaminated objects and inhalation of aerosols. Indeed, using guinea pig models of influenza infection, Mubareka et al. demonstrated that non infected guinea pigs were infected by infected guinea pigs at distances less than or equal to 80 to 100 cm [20]. In México City public transport is usually crowded, the underground (metro) carries 4.2 million people every day (http://www.setravi.df.gob.mx/reportajes/historia/historia.html), bringing people in close contact, and this may have facilitated sustained human-to-human transmission of the novel influenza A virus.

Three waves of influenza A (H1N1)v have been seen throughout México. Interestingly, a few days before the identification of the novel virus on April 23th, 2009, a mass gathering took place in Mexico during the Easter season (April 5th to 11th), known as Semana Santa (Holy Week). In this mass event, 2 million additional people gather in Iztapalapa to observe the Iztapalapa Passion Play, a representation of the crucifixion of Jesus [21]. At this time however, this congregation of people may have contributed to the spread of the virus during the initial phase of the first wave, with individuals who had acquired the influenza A (H1N1)v, disseminating the virus to others attending the Iztapalapa Passion Play, who in turn spread this infection throughout Mexico City and beyond. In addition, infected individuals returning to México City at the end of the Semana Santa holiday in places such as Veracruz and Oaxaca may have also contributed to dissemination of the novel virus throughout the City. Several reports have highlighted the importance of undertaking continual vigilance to improve public health and prevent the transmission of the pandemic influenza A (H1N1)v during mass gatherings [22]–[24]. A second wave however, had the largest numbers of confirmed influenza cases located in Southern Mexico, mostly within the states of Chiapas Quintana Roo and Yucatan which coincided with the summer school holiday. This pattern of distribution is consistent with the view that travellers from Mexico City to the most popular tourist destination in South of Mexico (Cancun) could have contributed to the spread of the virus during the summer holiday. Both the Association of Hotels of Cancun [personal communication]) and Leyva de la Cruz [25] estimated that 776,250 travellers visited Cancun between June and August 2009 (47% were Mexicans: 28% travelled from Mexico City) which represents 1293 visitors/1000 local inhabitants. It is well established that human mobility plays an important role in disease transmission [26], [27]. Interestingly, Lipsitch et al have previously proposed that foreign travellers who had visited Mexico could have spread the novel virus worldwide (Cancun accounted for 45–74.5% of visitors depending on the nationality) [28]. In this last study, authors reported an attack rate of 12.1/100.000 in Quintana Roo during the first wave (as May the 9^th^). In contrast we have estimated an attack rate of 42.4/100.000. Discrepancies between these studies could be explained because calculations in the study conducted by Lipsitch el al were made early during the outbreak of the virus when many cases had not been fully confirmed. In September a third wave began with a greater case-fatality rate which remained highly active until the end of December. Deaths occurred predominantly in patients aged 30–59 years (53% of total deaths) and affected pregnant women and patients with underlying conditions such as diabetes and obesity [29], [ 30]. It should be noted that the beginning of the third wave coincided with the start of the school year in Mexico which in average include 34 million students (from elementary school to University students) throughout the Country (www.presidencia.gob.mx/prensa/?contenido=55183). The massive number of students returning to schools could have contributed to the transmission of the virus during this third wave of activity.

Our analysis has some potential limitations. First, part of our assumptions were made on published epidemiological data which could show some heterogeneity on the data collected from the different Mexican States including different clinic definitions, timing of sample collection and the sample size may be underestimated as asymptomatic- and mild cases did not require medical care. Secondly, although we have provided information about travelers to Cancun we did not analyze data from other Cities within Quintana Roo neither investigated another South states affected by the influenza A (H1N1)v. And thirdly, we have postulated that the congregation of people which took place in Iztapalapa Passion Play contributed to the spread of the virus. However future studies need to be undertaken to provide an estimated analysis of the virus transmission during this mass gathering.

In summary, we have shown that in the initial stages of the influenza A (H1N1) virus outbreak, the majority of infected patients taking part in this study lived in highly populated areas of Mexico City and most of them developed mild to moderate respiratory symptoms affecting predominately young people. Subsequently, we have shown that the virus spread to the entire country with three waves of activity between April and December 2009. Interestingly, a mass gathering took place in Mexico (the Iztapalapa Play Passion) and we postulate that it may have contributed to the spread of the novel virus. Because gatherings contribute to an increase in the transmission of infectious diseases, appropriate contingency plans to reduce substantially the impact of influenza virus infection should be undertaken. These include surveillance, provision of sufficient stocks of anti-viral drugs, vaccination and early detection of H1N1 strains, particularly as novel influenza A (H1N1) virus mutants are emerging in different parts of the world [9], [31].

## Supporting Information

Figure S1 Clinical characteristic of Patients. A. Symptoms of patients (n = 202) with confirmed cases of human infection with 2009 influenza A (H1N1)v. B. Age groups of patients (n = 202) who were infected with 2009 influenza A (H1N1)v.
(0.18 MB TIF)
