# Supplementary figures and images for: Inside the Outbreak of the 2009 Influenza A (H1N1)v Virus in Mexico
Source: PLoS One. 2010 Oct 8;5(10):e13256. doi: 10.1371/journal.pone.0013256 (PMC2951908; doi:10.1371/journal.pone.0013256)

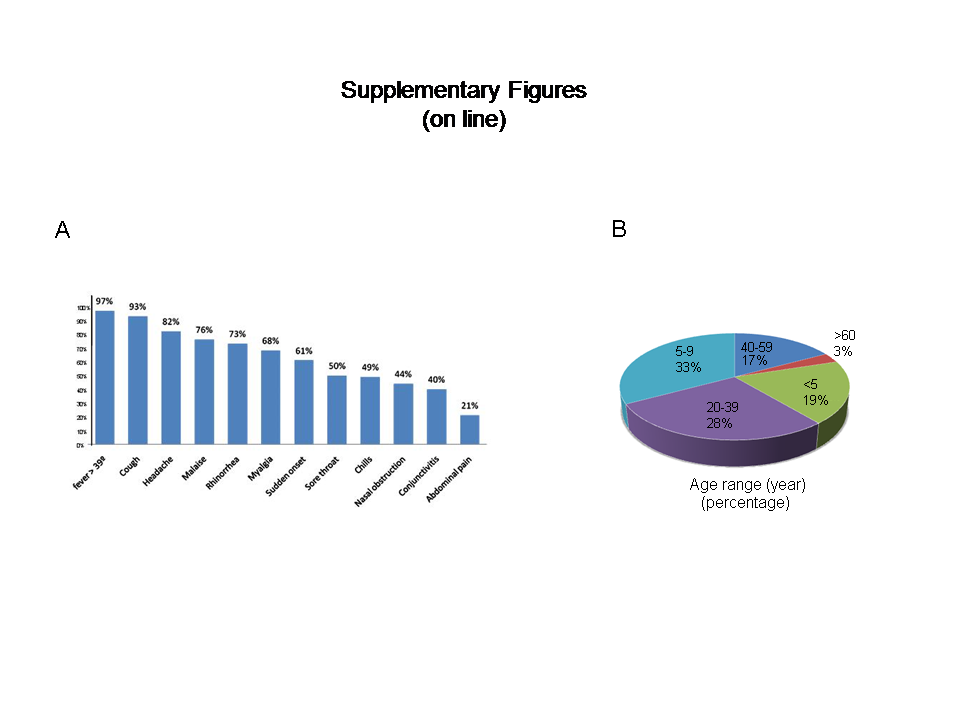

Supplement: Figure S1 — Clinical characteristic of Patients. A. Symptoms of patients (n = 202) with confirmed cases of human infection with 2009 influenza A (H1N1)v. B. Age groups of patients (n = 202) who were infected with 2009 influenza A (H1N1)v. (0.18 MB TIF) [file pone.0013256.s001.tif]
